# Reference Genes Selection for Quantitative Real-Time PCR Using RankAggreg Method in Different Tissues of *Capra hircus*

**DOI:** 10.1371/journal.pone.0083041

**Published:** 2013-12-16

**Authors:** Mohammad Javad Najafpanah, Mostafa Sadeghi, Mohammad Reza Bakhtiarizadeh

**Affiliations:** 1 Department of Animal Science, College of Agriculture and Natural Resources, University of Tehran, Karaj, Iran; 2 Department of Animal and Poultry Science, College of Aburaihan, University of Tehran, Tehran, Iran; Institute of Farm Animal Genetics, Germany

## Abstract

Identification of reference genes with stable levels of gene expression is an important prerequisite for obtaining reliable results in analysis of gene expression data using quantitative real time PCR (RT-qPCR). Since the underlying assumption of reference genes is that expressed at the exact same level in all sample types, in this study, we evaluated the expression stability of nine most commonly used endogenous controls (GAPDH, ACTB, 18S rRNA, RPS18, HSP-90, ALAS, HMBS, ACAC, and B2M) in four different tissues of the domestic goat, *Capra hircus*, including liver, visceral, subcutaneous fat and longissimus muscles, across different experimental treatments (a standard diet prepared using the NRC computer software as control and the same diet plus one mg chromium/day). We used six different software programs for ranking of reference genes and found that individual rankings of the genes differed among them. Additionally, there was a significant difference in ranking patterns of the studied genes among different tissues. A rank aggregation method was applied to combine the ranking lists of the six programs to a consensus ranking. Our results revealed that HSP-90 was nearly always among the two most stable genes in all studied tissues. Therefore, it is recommended for accurate normalization of RT-qPCR data in goats, while GAPDH, ACTB, and RPS18 showed the most varied expressions and should be avoided as reference genes.

## Introduction

Over 830 million of the domestic goats, *Capra hircus*, belonging to more than 1000 breeds are reared throughout the world because of their importance as sources of meat, milk, fiber and pelts (http://www.fao.org/corp/statistics/en). In addition to their value as domestic animals, goats have been extensively used as animal models for biomedical studies, to investigate the genetic basis of complex traits as well as in the transgene production of peptide medicines[1,2].The recent report of whole genome sequence of the domestic goat [1] has led to a striking increase in its use as a model species in a wide range of genetic studies including gene expression analyses.

Quantitative real-time PCR (RT-qPCR) is the most widely used method for gene expression studies and analysis of biological pathways, as it allows fast, extremely sensitive, and highly reproducible quantification of mRNA levels [3-5]. However, some factors including RNA stability, RNA extraction, retrotransciption efficiency, PCR steps, and amounts of RNA added into the reaction may negatively affect the accuracy and reliability of the results obtained from RT-qPCR [6,7]. Several procedures have been developed for normalization of the variations from sample to sample among which, the most common and reliable method is to normalize the total amounts of RNA or a single internal reference gene, known as housekeeping gene [8,9]. An ideal reference gene is expected to be stable in terms of expression level across various experimental conditions such as developmental stages, tissue types, experimental treatments, and external stimuli [10]. However, recent studies have shown that many commonly used reference genes are not suitable for RT-qPCR, as their expression might be altered by some experimental conditions [11-13]. Therefore, it is essential to screen and select appropriate reference gene(s) with a constant level of expression under certain experimental conditions for valid interpretation of expression data [14].

To date, several mathematical models and software programs have been developed which allows selection of the most stable reference genes [5,7,13,14]. However, it has been repeatedly reported that different software programs may give different rankings of reference genes [15-17]. This may be explained by the fact that the routine programs use different algorithms, thus they would not be expected to yield identical results. Recently, a rank aggregation method was developed to combine the ordered lists of genes, obtained from different software programs, to a consensus ranking of reference genes [15-20]. 

The objective of this study was to select from a panel of nine commonly used reference genes the most stable genes for normalization of gene expression data in the domestic goat, *C. hircus*. Six different software programs were used to identify the most stable reference genes across the four studied tissues under chromium treatment. Additionally, a rank aggregation method was used to provide a consensus ranking by combining the ranking results obtained from six different programs. The variant experimental conditions include four different tissue types (liver, visceral fat, subcutaneous fat, and longissimus lumborum (LL) muscles) as well as two dietary conditions based on presence or absence of the supplementary trivalent chromium (Cr^+3^). Trivalent chromium has been known as an essential element for normal metabolism of proteins, lipids, carbohydrates, and nucleic acids in human and husbandry animals such as cows, sheep, and goats[21,22]. A large body of evidence has also confirmed the positive effects of chromium on various biological properties of domestic animals including immunity against viral diseases [23], live body weight, daily weight gain, dressing percentage, longissimus muscle area, nutrient digestibility, etc. [21,22,24,25]. Importantly, chromium has been suggested to alter the expression level of a variety of genes in human and laboratory animals [26-28]. Therefore, we used the trivalent chromium as food supplementation to evaluate the effects of external agents on expression stability of the nine candidate genes.

## Materials and Methods

### Ethics Statement

All experiments with animals were performed according to the recommendations in the Guide for the Care and Use of Laboratory Animals of the Research Station of Department of Animal Science, University of Tehran, Iran. The protocols were approved by the Animal Care and Use Committee of the University of Tehran Institutional Animal Care and Use Committee and included in a Research project.

### Animal husbandry and experimental design

This study was conducted at the Research Station of Department of Animal Science, College of Agriculture and Natural Resources, University of Tehran, Iran. Twenty-four, 4 to 5-months old male goat kids belonging to the native Iranian breed, Mahabadi, were selected for the experiment. All procedures of immunity and nutrition were conducted under protocols approved by this station.

The kids were weighed (BW=22 ± 2 kg) and allocated randomly to one of the two following dietary treatments: standard diet plus 0 and 1 mg chromium per day as chromium-methionine (Availa®Cr 1000, Zinpro Corporation, USA). The standard diet was balanced and prepared using NRC computer software (Table 1). The kids were individually penned for 100 days (10 days for adaptation and 90 days for feeding period), with access to enough water, and provided with the prepared diets twice a day (08:00 h and 17:00 h). The kids were weighed before the morning feeding meal triweekly (i.e. after 14-16 hours of starvation) throughout the experimental period to determine changes in their body weight. 

**Table 1:** Ingredient and chemical composition of basal standard diet fed to goat kids.

Ingredient	% of DM
Alfalfa hay	16.49
Corn silage	8.32
Wheat straw	5.19
Barley grain	51
Wheat bran	9.09
Canola meal	4.55
Soybean meal	2.21
Calcium carbonate	1.3
Mineral-vitamin supplement ^a^	0.91
Sodium bicarbonate	0.78
Salt	0.52
Nutrient fractions	
DM (%)	80.78
CP (% of DM)	13.5
Ether extract (% of DM)	2.6
NDF (% of DM)	36.6
Ash (% of DM)	9
ME (Mcal/kgDM)	36.6
Calcium (% of DM)	0.89
Phosphorus (% of DM)	0.49
Chromium (% of DM)	0.83

^a^ Containing per kg DM: calcium, 195 g; phosphor, 80 g; magnesium, 21000 mg; sodium, 50 g; manganese, 2200 mg; iron, 3000 mg; copper, 300 mg; iodine, 120 mg; cobalt, 100 mg; zinc, 300 mg; selenium, 1.1 mg; antioxidant, 2500 mg; vitamin A, 600,000 IU; Vitamin D3, 200,000 IU; vitamin E, 200 mg.

### Slaughtering and tissue sampling

After feeding on the prepared diets for 90 days, the kids were transferred to the departmental abattoir, where they were kept for 12 h with free access to water. They were then slaughtered by decapitation and tissue samples from liver, visceral fat, subcutaneous fat, and longissimus lumborum muscle were taken from the corpses. The samples were immediately frozen in liquid nitrogen and transferred to the laboratory, where they were maintained at -80 °C until used. 

### Total RNA isolation, clean up and cDNA synthesis

Total RNA was extracted according to the method of Chomczynski and Sacchi (2006) using Trizol Reagent (Invitrogen Co., Carlsbad,CA, USA) [29]. The extracted RNA was then treated with RNase-free DNase I (TaKaRa, Shuzo, Kyoto, Japan). RNA concentrations were estimated by Nanodrop spectrophotometry at 260 nm and their purities were checked by determining the absorption ratios at 260/280 nm. The quality of extracted RNA was assessed by electrophoresis at 1% agarose-gel containing Ethidium Bromide. First-strand cDNA was synthesized from 100 ng of total RNA using an oligo (dT) primer, random hexamers and a commercially available kit (AccuPower^®^ RocketScript^™^ RT PreMix) according to manufacturer’s instructions.

### Selection of reference genes and primer designing

Nine classical reference genes including glyceraldehyd-3-phosphate dehydrogenase (GAPDH), beta-actin (ACTB), heat shock protein-90 (HSP-90), aminolevulinate synthase 1 (ALAS1), 18S ribosomal RNA (18S rRNA), ribosomal protein S18 (RPS18), hydroxyl methyl bilane synthase (HMBS), acetyl coenzyme A carboxylase alpha (ACAC-α), and beta-2-microglobulin (B2M) were considered as candidate genes. The nucleotide sequences of all candidate genes (except for B2M) belonging to the domestic goat (*Capra hircus*) were obtained from the GenBank database (http://www.ncbi.nlm.nih.gov). For B2M gene, only one sequence was available for the relative species, *Ovis aries*. Primer pairs were designed from these sequences (optimal T_m_ at 59.8°C and GC% between 45-50%) using primer3Plus online software [30] and checked using OligoAnalyzer 3.1 (http://eu.idtdna.com/analyzer/applications/oligoanalyzer/), OligoCalc [31], and PrimerBLAST [32] (Table 2).

**Table 2:** Sequence and some characterization of specific primer pairs for the nine genes used for selection of reference gene in goat kids.

Gene Name	Accession number	Sense primer sequence 5ʹ→3ʹ	Anti-sense primer sequence 5ʹ→3ʹ	Length (bp)	T_m_ (°C)
GAPDH	AJ431207.1	GGCACAGTCAAGGCAGAGAA	TCTCGCTCCTGGAAGATGGT	71	59.8
ACTB	HQ993072.1	GCAAGGACCTTTACGCCAAC	CTTGATCTTCATCGTGCTGGG	116	59.8
HSP-90	AF548366.1	GCCCGAGATAGAAGACGTTG	AGTCGTTGGTCAGGCTCTTG	197	59.8
ALAS	AB232536.1	ATGTGGCCCACGAGTTTGG	CTTGTGCTGGCGATGTACC	178	59.8
18S rRNA	DQ149973.1	TAATCCCGCCGAACCCCATT	GGTGTGTACAAAGGGCAGG	125	59.8
RPS18	EF564275.1	ATGCAGAATCCACGCCAATAC	GGCCCGAATCTTCTTCAGG	147	59.8
HMBS	AB232537.1	CTTGCCAGAGAAGAGTGTGG	CAGCCGTGTGTTGAGGTTTC	115	59.8
ACAC	DQ370054.1	CGCTATGGAAGTCGGCTGTG	CAGGAAGAGGCGGATGGGAA	105	59.8
B2M	DQ386890.1	TGTCCCACGCTGAGTTCACT	TGAGGCATCGTCAGACCTTGA	137	59.8

### Two step real-time RT-PCR

Real-time Quantitative PCR was performed using SYBR Green I technology on iQ5 System (BioRad, USA). The reactions consisted of 1x SYBR Green PCR Master Mix (SYBR biopars, GUASNR, Iran), 300 nm of each specific forward and reverse primers, 10 ng of cDNA, and nuclease free water to a final volume of 20 µL. 

The cycling conditions were as follows: cDNA was denatured at 94 °C for 3 min, followed by 35 cycles of 94 °C for 15 s and 59.8 °C for 15 s (gain set at 10 for SYBR Green). All samples were amplified in triplicate from the same RNA preparation and the mean value was considered. Two biological replications were used for each plate. The real-time RT-qPCR efficiency was assessed for each gene based on the slope of a linear regression model [33]. The bulks of each cDNA sample were used as PCR template in a range of 10-fold dilution series. The corresponding real-time RT-qPCR efficiencies were calculated based on the slope of the standard curve using the following equation: (E = 10 ^-1/slope^ - 1) [34].

### Determination of genes expression stability

Six popular software programs including geNorm version 2.3 [15], NormFinder version 0.953 [10] and BestKeeper version 1 [35], qBasePlus version 2.3 (Biogazelle, Ghent, Belgium) and GenEx version 5 (NormFinder and GeNorm) were used to validate the most stable reference genes for different tissues taken from goat kids. In general, all programs are based on the principle that the expression of reference genes should be stable under all experimental conditions and tissue types studied. 

### Statistical Method for Rank Aggregation

The RankAggreg package of R software was used to combine the stability measurements obtained from the six software and establish a consensus rank of reference genes [36]. The term stability refers to the variation of the Ct values for a given gene across different samples or experimental conditions. Transcripts with the lowest stability measurement would usually yield the best reference gene. Six matrix of rank-ordered reference genes, according to the different stability measurements (NormFinder stability value, M values by qBasePlus, geNorm (qbase plus) and GenEx (geNorm), standard deviation of BestKeeper and GenEx (NormFinder) were used as input for this statistical package (for each of the studied tissues separately and all tissues together). An unweighted rank aggregation was applied by using BruteAggreg function of the package. This function performs rank aggregation using the brute force approach. 

The aim of rank aggregation is to ﬁnd an aggregated ranking that minimizes the distance to each of the ranked lists in the input set. The distance among ordered lists is calculated using the Spearman foot rule function. In summary Brute force approach was used to account all ranking lists and find the list with the minimum Spearman foot rule distance according to the Brute Aggreg function. 

The permutation function of the gtools package was used to generate all possible ordered lists. This approach is suggested when the size of the ranking list is smaller than10. RankAggreg function is another function of this package that performs rank aggregation via the Cross-Entropy Monte Carlo algorithm. This algorithm is usually recommended when the size of the ranking list is larger than 10 [36]. Since, our size of the ranking list was near to 10 (9), we used RankAggreg function additionally to validate the consensus rank of reference genes resulting from the brute force approach. Because GenEx (geNorm) yields the same M stability value as the two most stable genes, two consensus lists of reference genes were created by altering the position of the two most stable genes. The consensus ranking with the lower score was selected.

## Results

**Figure 1:**
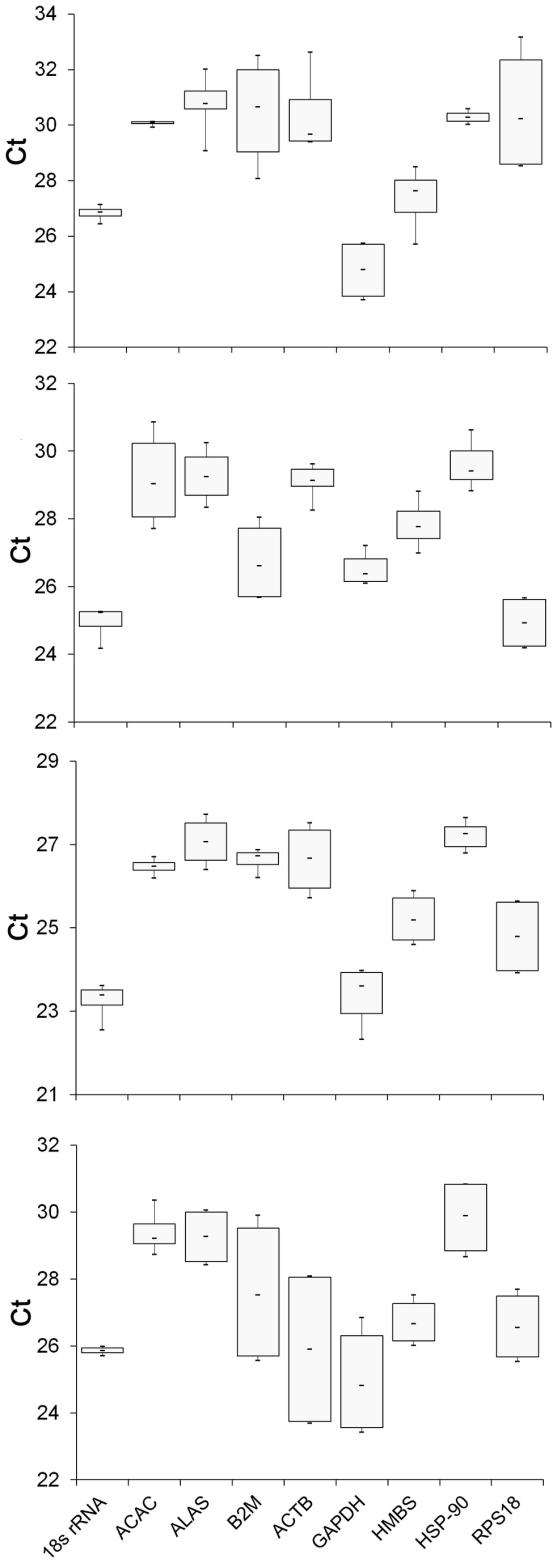
The distribution of gene expression levels of nine candidate reference genes analyzed for four different tissues (liver (a), visceral fat (b), subcutaneous muscle (c), and longissimus muscle (d)) in pooled C_T_ value.

**Figure 2:**
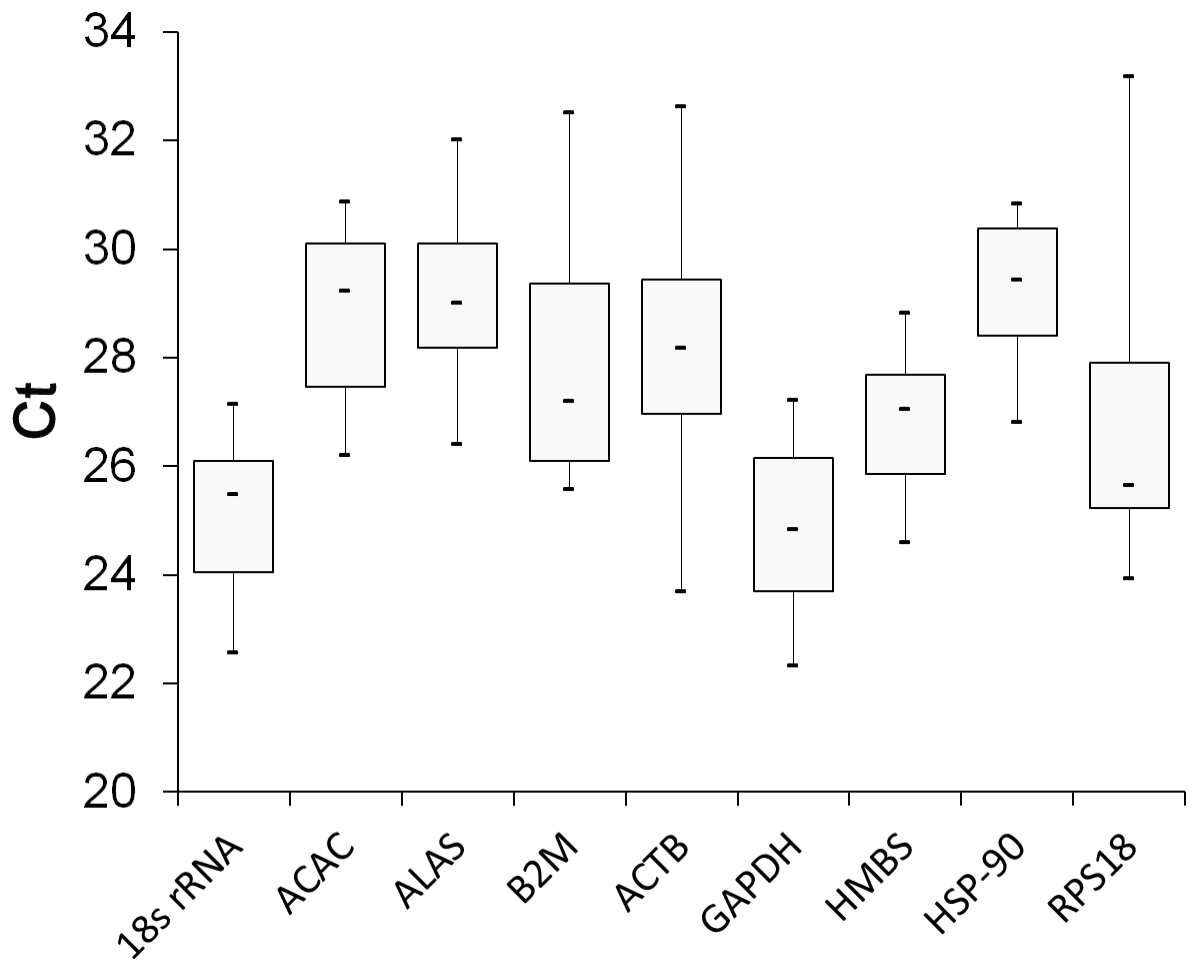
The distribution of gene expression levels of nine candidate reference genes in pooled C_T_ value. The variations are related to data taken together from four different tissues (liver, visceral fat, subcutaneous muscle, and longissimus muscle).

In this study, after a detailed literature review, nine reference genes, including GAPDH, ACTB, HSP-90, ALAS1, 18S rRNA, RPS18, HMBS, ACAC-α and B2M were used to select a suitable reference gene for expression analyses in different tissues of domestic goats. These genes are among the most common ones frequently used as reference genes for normalization of RT-qPCR data across animal taxa. To conﬁrm reproducibility of real-time PCR, standard error of means (SEM) was determined (Table 3). We found a wide variation in the averages of the cycle threshold (C_T_) values for the nine reference genes where it ranged between 23.71 to 33.17 in liver, 24.18 to 30.86 in visceral fat, 22.33 to 27.72 in subcutaneous fat, and 23.42 to 30.84 in longissimus lumborum muscle tissues (these values has been visualized in Figure 1 for each studied tissue and in Figure 2 for all tissues together). The highest and lowest expression levels were detected for 18S rRNA in subcutaneous fat and for ALAS in liver, respectively. Although, the expression of the studied genes showed some degrees of differences across different tissues and dietary treatments, the most stability was recorded for HSP-90 (Figure 2). This was implied by the fact that HSP-90 was nearly always among the most three stable genes ranked by the six programs for all tissues (Tables 4-7). The GAPDH, RPS18 and ACTB genes, in contrast, were among the most unstable genes in term of expression level (Tables 4-7). Table 8 shows the ranking of the nine candidate genes by considering all sample tissues as a single unit (Information S1). 

**Table 3:** The calculated mean the cycle threshold (C_t_) values and their SEM for the nine reference genes in different tissues.

Reference gene	Treatment	Tissue			
		Liver	Visceral Fat	Subcutaneous Fat	LL Muscle
HSP-90	Control	30.47 ± 0.067	29.06 ± 0.116	27.11 ± 0.179	30.83 ± 0.009
	Chromium	30.13 ± 0.055	30.13 ± 0.283	27.33 ± 0.223	28.81 ± 0.087
ALAS	Control	31.37 ± 0.378	28.63 ± 0.167	26.58 ± 0.104	30.01 ± 0.032
	Chromium	30.15 ± 0.544	29.91 ± 0.196	27.56 ± 0.095	28.50 ± 0.046
B2M	Control	32.10 ± 0.237	25.70 ± 0.012	26.82 ± 0.038	29.60 ± 0.179
	Chromium	29.43 ± 0.450	27.79 ± 0.153	26.46 ± 0.144	25.67 ± 0.058
RPS18	Control	28.85 ± 0.381	24.23 ± 0.023	25.62 ± 0.012	27.53 ± 0.095
	Chromium	28.58 ± 0.029	25.63 ± 0.026	23.96 ± 0.023	25.65 ± 0.061
GAPDH	Control	23.82 ± 0.061	26.14 ± 0.020	22.83 ± 0.286	26.42 ± 0.251
	Chromium	25.72 ± 0.017	26.90 ± 0.185	23.94 ± 0.023	23.54 ± 0.066
ACAC	Control	30.12 ± 0.009	27.99 ± 0.153	26.36 ± 0.092	29.01 ± 0.156
	Chromium	30.04 ± 0.061	30.35 ± 0.294	26.58 ± 0.078	29.76 ± 0.346
ACTB	Control	31.27 ± 0.785	28.94 ± 0.393	25.91 ± 0.110	28.07 ± 0.014
	Chromium	29.43 ± 0.012	29.27 ± 0.147	27.38 ± 0.081	23.74 ± 0.026
18s rRNA	Control	26.71 ± 0.153	25.27 ± 0.003	23.44 ± 0.058	25.85 ± 0.084
	Chromium	26.97 ± 0.101	24.70 ± 0.300	23.09 ± 0.306	25.87 ± 0.052
HMBS	Control	28.12 ± 0.222	27.33 ± 0.196	24.69 ± 0.052	27.31 ± 0.121
	Chromium	26.64 ± 0.534	28.35 ± 0.274	25.75 ± 0.081	26.12 ± 0.064

**Table 4:** Results of ranking of nine candidate reference genes obtained using six different software programs and rank aggregation of candidate reference genes for liver.

Rank Position	GenEx (NormFinder)	GenEx (geNorm)	NormFinder	BestKeeper	geNorm	qBasePlus	Consensus
1	HSP-90	HSP-90	HSP-90	ACAC	ACAC^a^	ALAS	HSP-90
2	ACAC	ACAC	ACAC	HSP-90	HSP-90	HMBS	ACAC
3	HMBS	18s rRNA	HMBS	18s rRNA	18s rRNA	HSP-90	18s rRNA
4	ALAS	ALAS	ALAS	ALAS	ALAS	ACTB	ALAS
5	18s rRNA	HMBS	18s rRNA	HMBS	HMBS	ACAC	HMBS
6	ACTB	ACTB	ACTB	GAPDH	ACTB	18s rRNA	ACTB
7	B2M	B2M	B2M	ACTB	B2M	B2M	B2M
8	RPS18	RPS18	RPS18	B2M	RPS18	RPS18	RPS18
9	GAPDH	GAPDH	GAPDH	RPS18	GAPDH	GAPDH	GAPDH

^a^ ACAC was the first in consensus list if alter the position of the two most stable genes in the geNorm list.

**Table 5:** Results of ranking of nine candidate reference genes obtained using six different software programs and rank aggregation of candidate reference genes for visceral fat.

Rank Position	GenEx (NormFinder)	GenEx (geNorm)	NormFinder	BestKeeper	geNorm	qBasePlus	Consensus
1	RPS18	ALAS	ALAS	18s rRNA	HSP-90	HSP-90	HSP-90
2	ALAS	HMBS	HSP-90	ACTB	HMBS	HMBS	HMBS
3	HSP-90	HSP-90	GAPDH	GAPDH	ALAS	ALAS	ALAS
4	HMBS	RPS18	HMBS	HMBS	RPS18	RPS18	RPS18
5	GAPDH	GAPDH	RPS18	HSP-90	GAPDH	GAPDH	GAPDH
6	B2M	B2M	ACTB	ALAS	ACTB	ACTB	ACTB
7	ACTB	ACAC	B2M	RPS18	B2M	B2M	B2M
8	ACAC	ACTB	ACAC	B2M	ACAC	ACAC	ACAC
9	18s rRNA	18s rRNA	18s rRNA	ACAC	18s rRNA	18s rRNA	18s rRNA

**Table 6:** Results of ranking of nine candidate reference genes obtained using six different software programs and rank aggregation of candidate reference genes for subcutaneous fat.

Rank Position	GenEx (NormFinder)	GenEx (geNorm)	NormFinder	BestKeeper	geNorm	qBasePlus	Consensus
1	ALAS	HSP-90	ALAS	HMBS	HSP-90	ALAS	ALAS
2	HSP-90	ALAS	HSP-90	18s rRNA	ALAS	HSP-90	HSP-90
3	HMBS	ACAC	HMBS	HSP-90	HMBS	HMBS	HMBS
4	18s rRNA	18s rRNA	ACAC	ALAS	ACAC	18s rRNA	18s rRNA
5	ACAC	HMBS	18s rRNA	ACAC	B2M	ACAC	ACAC
6	B2M	GAPDH	B2M	GAPDH	18s rRNA	B2M	B2M
7	GAPDH	B2M	GAPDH	ACTB	GAPDH	GAPDH	GAPDH
8	ACTB	ACTB	ACTB	B2M	ACTB	ACTB	ACTB
9	RPS18	RPS18	RPS18	RPS18	RPS18	RPS18	RPS18

**Table 7:** Results of ranking of nine candidate reference genes obtained using six different software programs and rank aggregation of candidate reference genes for longissimus muscle.

Rank Position	GenEx (NormFinder)	GenEx (geNorm)	NormFinder	BestKeeper	geNorm	qBasePlus	Consensus
1	ACAC	ALAS	ACAC	ACAC	HMBS	HSP-90	ACAC
2	HSP-90	GAPDH	HSP-90	B2M	GAPDH	ACAC	HSP-90
3	B2M	ACTB	B2M	HSP-90	ALAS	ALAS	ALAS
4	ALAS	HMBS	ALAS	18s rRNA	HSP-90	HMBS	B2M
5	HMBS	ACAC	HMBS	ALAS	ACAC	GAPDH	HMBS
6	18s rRNA	HSP-90	18s rRNA	HMBS	18s rRNA	18s rRNA	18s rRNA
7	GAPDH	B2M	GAPDH	GAPDH	B2M	B2M	GAPDH
8	ACTB	18s rRNA	ACTB	ACTB	ACTB	ACTB	ACTB
9	RPS18	RPS18	RPS18	RPS18	RPS18	RPS18	RPS18

**Table 8:** Results of ranking of nine candidate reference genes obtained using six different software programs and rank aggregation of candidate reference genes for all studied tissues.

Rank Position	GenEx (NormFinder)	GenEx (geNorm)	NormFinder	BestKeeper	geNorm	qBasePlus	Consensus
1	ALAS	HSP-90	ALAS	HMBS	HSP-90	ALAS	ALAS
2	HSP-90	ALAS	HSP-90	18s rRNA	ALAS	HSP-90	HSP-90
3	HMBS	ACAC	HMBS	HSP-90	HMBS	HMBS	HMBS
4	18s rRNA	18s rRNA	ACAC	ALAS	ACAC	18s rRNA	18s rRNA
5	ACAC	HMBS	18s rRNA	ACAC	B2M	ACAC	ACAC
6	B2M	GAPDH	B2M	GAPDH	18s rRNA	B2M	B2M
7	GAPDH	B2M	GAPDH	ACT-B	GAPDH	GAPDH	GAPDH
8	ACT-B	ACT-B	ACT-B	B2M	ACT-B	ACT-B	ACT-B
9	RPS18	RPS18	RPS18	RPS18	RPS18	RPS18	RPS18

The BestKeeper software uses a pairwise correlation analysis of all reference genes and calculates the geometric mean of the best suited ones. BestKeeper uses two most important criteria including the stability (coefficient of variance (CV) and standard deviation (SD)) and coefficient of correlation to the BestKeeper index for evaluating the stability of reference genes. The genes with the lowest CV and SD are considered as the most stable genes [37,38]. Results based on BestKeeper showed that the most stable gene in both liver and subcutaneous fat under chromium treatment was ACAC. But in visceral fat and longissimus lumborum muscles 18srRNA was identified as the most stable gene. HMBS and 18srRNA were found to have a remarkably stable expression in all studied tissues together.

GeNorm, the ﬁrst reported software, is based on pairwise comparison model and calculates the M value, where M is the average pairwise variation of an individual gene to other genes, of all candidate genes and use geometric averaging across a matrix of reference genes [15,17]. Genes are ranked according to their expression stability by a repeated process of stepwise exclusion of the least stably expressed genes [39]. Via geNorm, M values of all reference genes were less than 1.5 indicating that these candidate genes have stable expression levels. Among nine reference genes, the most stable genes in liver were ACAC and HSP-90, in visceral fat were HSP-90 and HMBS and in LL muscles were HMBS and GAPDH. But in subcutaneous fat and all studied tissues together, ALAS and HSP-90 were identified as most stable reference genes.

The qBasePlus evaluates the stability of the applied reference genes by calculating two quality measures: the coefficient of variation (CV) and the geNorm stability M-value. Both values are only meaningful, or can be calculated only if multiple reference genes are quantified. The reference genes with the lower quality values would have the higher stability [40]. The algorithm of qBasePlus for calculation of relative quantities selecting different reference genes and specific efficiencies has four steps: 1) Calculation of the average C_t_ value for all replicates of the same gene/sample combination within a given run, 2) transformation of mean C_t_ value into relative quantity using the gene specific PCR efficiency, 3) calculation of the normalization factor and 4) calculation of the normalized relative quantity for gene of interest for each sample [40]. Our results of qBasePlus revealed that the most stable gene in liver, subcutaneous fat and all studied tissues together under chromium treatment was ALAS, whereas in visceral fat and LL muscle tissues was HSP-90. 

NormFinder software program employs an ANOVA-based model to estimate overall reference gene stability; but also considers variations between sample subgroups. The software calculates a stability value for all candidate reference genes. The stability value is based on the combined estimate of intra- and inter-group expression variations of the studied genes [41,42]. Using NormFinder, the top-ranked reference gene was ALAS in visceral and subcutaneous fat tissues and all studied tissues together. NormFinder also indicates HSP-90 and ACAC as the best stable reference genes for normalizing calculations in Liver and LL muscles, respectively. 

GenEx (http://www.multid.se/) has the advantage of incorporating both NormFinder and geNorm in the software. Thus, users can get both of these algorithms on which to base their choice of reference genes in one software installation [43]. These algorithms detect the most stably expressed genes in an experimental setup. Optimal number of reference gene was selected using pairwise variation analysis integrated in geNorm algorithm implanted in GenEx [44]. The accumulated standard deviation (Acc. SD), as an indicator for the optimal number of reference genes, was determined using GenEx software. For each sample, the normalization factor based on *n* reference genes was calculated as the geometric average of the *n* raw reference gene quantities [45]. The results of GenEx (NormFinder) shown that the most stable genes in liver, visceral and subcutaneous fat, LL muscles and all studied tissues together were HSP-90, RPS-18, ALAS, ACAC and ALAS, respectively. But in GenEx (geNorm) were HSP-90, ALAS, HSP-90, ALAS and HSP-90. 

We found significant differences in ranking patterns of reference genes obtained from different software programs (Tables 4-7). These discrepancies are probably related to differences in the algorithm each program uses in gene ranking. To provide a consensus ranking, a rank aggregation method was used to combine the ranking patterns of all six software. The RankAggreg package provides two functions for combining the ordered lists: the BruteAggreg function for k<10 and the RankAggreg function for k>10, where k is the size of the ranking list. In this study, the use of both functions yielded the same ranking list suggesting that the consensus ranks of genes were robust to the methods used. Additionally, the ranking lists were consistent when we altered the position of the two most stable genes in the geNorm list except for liver tissue. The results of rank aggregation method revealed that the two most stably expressed genes under chromium treatment were HSP-90 and ACAC in liver, HSP-90 and HMBS in visceral fat, ALAS and HSP-90 in subcutaneous muscles, and ACAC and HSP-90 in longissimus muscles (Tables 4-7). The three genes, GAPDH, RPS18 and ACTB showed the least stability in expression level across all studied tissues (Tables 4-7). The two candidate genes, ALAS and HSP-90 were nearly always the most stably expressed genes when different software programs were applied for the selection of suitable reference gene in all studied tissues (Tables 4-7). Therefore, it is not surprising that the rank aggregation method ranked the two genes as the most stable ones when compared to other candidate genes (Table 8).

## Discussion

Goats are of excellent economical and historical importance in Iran. Strong evidences suggest that the Fertile Crescent (stretching from the southern Levant in south eastern Turkey and northern Syria to the high Zagros Mountain pastures of Iran) is the center of domestication of both sheep and goat [46]. In addition to especial importance of goats as sources of milk, pelt, and meat, they have been long considered as a model species in a wide range of biological and medicinal studies [1,2]. Therefore, gene expression profiles of this animal in different tissues at the mRNA and protein levels would be extremely valuable for further elucidation of the molecular mechanisms involved in different biological pathways.

The RT-qPCR technique is one of the most common methods to understand gene expression profiles in different biological systems, which is an important step in identifying gene function. In this regard, the importance of reference genes to accurately analyze expression of a particular gene is well known. The best reference genes are expected to undergo a constant, unregulated expression in all experimental conditions and analyzed tissues. However, it has been clearly demonstrated that no universal reference gene exists that express stably in all experimental conditions [47,48]. To date, there are a few studies that use RT-qPCR as a technique for gene expression analyses in domestic goats. In most of these studies, the reference genes are usually selected based on literature review and experience in other organisms rather than empirical evidence in support of their efﬁcacy. But it is demonstrated that the most appropriate reference gene for animals of interest would not expect to be the same as found in other organisms, even if the animals are closely relative [49,50]. Therefore, it is generally proposed that the reference genes need to be validated for each species and for each specific experimental condition. 

Here, to improve the gene expression analysis by RT-qPCR in the domestic goats, we evaluated the suitability of nine candidate reference genes in four different tissues and under two different nutritional diets (presence or absence of chromium supplementation). Then, their expression was analyzed by using six different software programs: GeNorm, GenEx (GeNorm), qBasePlus, NormFinder, GenEx (NormFinder) and BestKeeper. 

The six software programs produced similar results, but the rankings were not identical and, in some cases, were substantially different, especially in the top ranked genes. Our results in this part suggest that different computer software might introduce a different ranking of reference genes. For example, the two genes, 18s rRNA and ACTB were among the least stable genes of visceral fat when the two programs GeNorm and NormFinder were applied, while, the program BestKeeper ranked these genes as the most stable ones (Table 5). Generally, the two programs GeNorm and NormFinder showed the most consistency compared with other studied software in term of ranking of the candidate reference genes (Tables 4-7). Difference in ranking results obtained from different software programs is a well-known criterion because the programs typically use different algorithms to determine gene expression stability [17-20]. The programs GeNorm, GenEx (GeNorm) and qBasePlus rank the reference genes according to a stability value (M). This value represents the mean pairwise variation between a candidate reference gene and all the other studied genes. The lowest M value indicates genes with the most stable expression. The genes are then ranked using stepwise elimination of the least stable genes [15,17]. qBasePlus also calculates a coefficient of variation (CV) for each gene as a stability measurement [17]. The NormFinder and GenEx (NormFinder) fit the data to a mathematical model, which allows comparison of intra- and intergroup variation and calculation of expression stability [10,17]. BestKeeper [33] uses repeated pairwise correlation analysis to determine the optimal reference genes. Therefore, it is not surprising that these algorithms differ in the ranking of the best reference genes.

Although our study was not [10,17] designed to measure the effect of chromium supplementation on individual reference gene expression, it appears that chromium affects the stability rankings of some specific genes. This implies that the expression of a given reference gene may vary with experimental conditions and should be tested in a set of conditions. In addition, our results revealed that the appropriate reference gene for a given tissue type may differ from those of other tissues. For example, the gene GAPDH was between the two most stable genes according to ranking results obtained from the GeNorm program for liver. However, in longissimus muscles, GAPDH was the worst candidate as reference gene (Tables 4 & 7). All of these findings strongly confirm the previously reported fact that different tissues may differ in the expression stability of the reference genes. 

We used rank aggregation method to combine the ordered lists of reference genes provided by different programs to a consensus rank. Results of rank aggregation using RankAggreg package offer that the most appropriate candidates as reference genes are HSP-90 for liver and visceral fat, ALAS for subcutaneous fat and ACAC for longissimus muscle (Tables 4-7). Generally, HSP-90 seems to be the most suitable candidate as reference gene across all studied tissues as it was nearly always among the two most stable genes (Tables 4-7). The suitability of HSP-90 as reference gene in expression studies has been previously confirmed in some other organisms [51]. Compared to the other reference genes examined, the three candidate genes GAPDH, ACTB, and RPS18 showed the least expression stability across all the studied tissues and are not appropriate enough for use as reference gene in gene expression analysis studies (Tables 4-7). Our results are in contrast to many other studies in which, GAPDH and ACTB have been reported to show high expression stability in different organisms (for examples see 52-54). 

To our knowledge, this is the ﬁrst study which investigates different candidate reference genes for gene expression analyses in four different tissues of the domestic goat (*C. hicus*) and seems to be useful in guiding researchers performing gene expression analyses in different breeds of this animal. Although, our results did not clarify any reference gene with constant expression level across all studied tissues, they highlighted HSP-90 as the most stably expressed gene that can be used for normalization of expression data in different tissues of the goat.

## Supporting Information

Information S1 **Threshold Cycle (Ct) of housekeeping genes in four tissues and original data of ranking (HKGs) via six softwares.**
(XLS)
